# Quality-Aware AIoT-Enabled Wireless Urban Sensor Networks Using Deep Learning

**DOI:** 10.3390/s26185825

**Published:** 2026-09-14

**Authors:** Carrow Morris-Wiltshire, Stuart Barr, Phil James, Tom Komar

**Affiliations:** School of Engineering, Newcastle University, Newcastle upon Tyne NE1 7RU, UK; stuart.barr@ncl.ac.uk (S.B.); philip.james@ncl.ac.uk (P.J.); tom.komar@ncl.ac.uk (T.K.)

**Keywords:** AIoT, wireless sensor networks, intelligent sensing systems, pedestrian monitoring, data quality, deep learning, movement prediction

## Abstract

This paper investigates the relationship between data quality and deep learning model performance for predicting pedestrian flows in smart cities, using data from the UK’s Newcastle Urban Observatory. We evaluate eight forecasting models (including multivariate LSTMs and Transformers) across 110 pedestrian sensors. Crucially, by preserving genuine data gaps, we reveal that the structure of missing data—specifically the length of consecutive sequences—is a more significant determinant of model performance than overall data completeness. Segmented regression identifies a sharp breakpoint at three hours of average sequence length, below which forecast error deteriorates rapidly. After controlling for sequence length, data completeness has no independent predictive power. Multivariate LSTMs and Transformers outperform all baselines, with Diebold–Mariano tests confirming statistical significance. A random forest meta-model successfully predicts per-sensor error ($R^{2}=0.81$) using basic quality metrics, highlighting sequence fragmentation as the dominant predictor. Finally, we show that sensors in transit-dense urban cores exhibit stronger diurnal signals and lower relative error, acting as spatial proxies for data quality. These findings establish key data quality requirements for forecasting urban dynamics and provide a framework for robust, quality-aware predictive systems.

## 1. Introduction

The Artificial Intelligence of Things (AIoT) and AIoT-enabled wireless sensor networks have become fundamental components of smart city infrastructure, enabling real-time monitoring and analysis of urban environments [1,2,3]. AIoT-enabled wireless sensor networks consist of spatially distributed autonomous sensors that cooperatively monitor physical or environmental conditions, transmitting data wirelessly to central gateways for processing and analysis [4]. Data quality in AIoT-enabled wireless sensor networks is critically important for making informed decisions about urban dynamics [5]. Current cities typically have limited decision-making functionality due to slow, incomplete, and largely disconnected information transmission systems [6]. However, as cities deploy more extensive networks of low-cost sensors to improve spatial resolution, they face increased data quality challenges [7]. Low-cost sensors are more prone to errors and inconsistencies than their expensive counterparts, and the volume of data generated makes manual quality assurance infeasible [8]. This necessitates automated systems that are ‘quality-aware’ and capable of assessing and managing data quality in real time [9,10].

While significant research exists on data quality for AIoT-enabled wireless sensor networks in environmental monitoring applications [10,11,12], there remains a distinct gap in understanding data quality requirements for complex dynamic systems such as urban mobility [13]. Urban mobility represents a complex adaptive system of systems, characterised by intricate interactions between pedestrian flows, transportation networks, and urban infrastructure [14]. The intellectual challenge lies in accurately predicting the spatio-temporal dynamics of these interconnected systems in real time—a task made particularly difficult by the non-linear nature of human movement patterns and the inherent variability in sensor data quality [5,7]. This predictive capability is a key enabling technology for urban digital twins, which promise to revolutionise city management through real-time emergency response, infrastructure maintenance optimisation, and resource monitoring [15,16].

**Figure 1:**
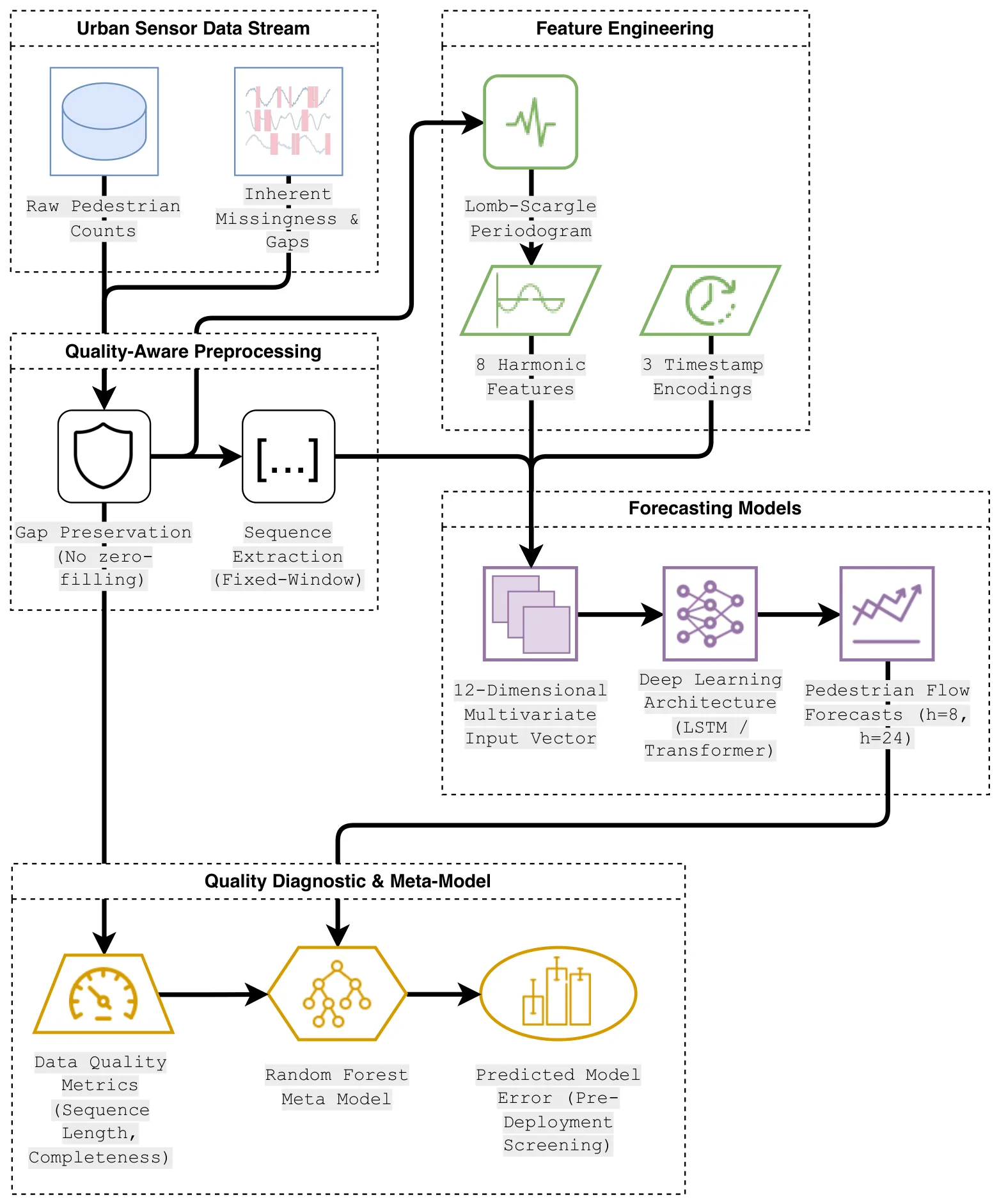
Overview of the proposed quality-aware prediction framework. The pipeline maps the progression from raw, gapped urban sensor data streams through gap-preserving preprocessing and feature engineering, culminating in deep learning forecasts. The empirical forecast error then serves as the target variable for the quality diagnostic meta-model.

The critical prerequisite for reliable prediction is understanding the degree to which the sensor network data can be trusted, requiring real-time ‘quality awareness’ of incoming data streams [8,17]. This paper begins to address this gap by investigating the relationship between data quality dimensions (different measures of data quality such as completeness) and the performance of a model predicting complex spatio-temporal dynamics of pedestrian movements [8]. The proposed quality-aware prediction framework is visually summarised in Figure 1.

The specific contributions of this paper are as follows. First, by preserving overnight data gaps as missing values rather than artificially imputing them as zeros on a dataset of 110 pedestrian sensors, we establish that the temporal structure of missing data, rather than its overall volume, governs forecast accuracy. Second, through segmented regression we identify a concrete data quality requirement: an average consecutive sequence length of approximately three hours (half a diurnal cycle) is needed before an LSTM can learn reliable temporal patterns. Third, we decouple the effects of data completeness and sequence fragmentation, showing via partial correlation and multiple regression that completeness only appears predictive because it co-varies with sequence length. Fourth, we quantify model superiority rigorously using Diebold–Mariano tests across all sensors. Finally, we connect data quality to the built environment, demonstrating that transit density and proximity to the urban core act as observable proxies for a sensor’s forecastability.

## 2. Literature Review

Current approaches to wireless sensor network data quality management reveal significant limitations when applied to urban mobility prediction. Traditional frameworks, such as those proposed by Wang et al. [18] and later refined by Karkouch et al. [7], emphasise static quality dimensions like accuracy and completeness but fail to adequately address the temporal dependencies crucial for movement prediction. While these frameworks provide valuable taxonomies for categorising data quality issues, they offer limited practical guidance for handling the dynamic nature of urban mobility data.

The limitations of existing approaches become particularly evident in real-time applications. Current quality control mechanisms, as reviewed by Teh et al. [8], typically rely on post hoc detection and correction of data quality issues, making them unsuitable for real-time decision support systems. Furthermore, most existing studies have focused on synthetic datasets or controlled environments [19,20], leaving a significant gap in understanding how these approaches perform with the messy, incomplete data streams characteristic of real-world urban sensor networks. This gap is particularly problematic for pedestrian monitoring systems, where data quality issues can arise from various sources including environmental conditions, sensor malfunctions, and communication failures [17]. While prior work on pedestrian flow forecasting often assumes relatively continuous data streams [21], real-world deployments are frequently plagued by missing data and temporal fragmentation. Studies specifically examining the impact of missing or fragmented sensor data on predictive performance remain scarce, with most literature defaulting to simple imputation strategies without rigorously quantifying how the structural nature of missingness (e.g., gap length) constrains deep learning models. Our work bridges this gap by explicitly decoupling data completeness from sequence fragmentation and directly measuring their impacts on prediction accuracy.

Teh et al. finds that the most common approaches to error detection and fault correction use artificial neural networks [8]. Aouedi et al. find that traditional methods such autoregressive integrated moving average (ARIMA) struggle to model the non-linearity and complex dependencies often found in traffic data, leading to lower prediction accuracy, especially for longer-term forecasting [21]. Liu et al. compare performance of deep learning models for traffic forecasting, including LSTMs and transformers [22]. They find that while transformers achieved higher prediction accuracy, they generally have a larger number of parameters (millions) compared to LSTMs (hundreds of thousands), stating that transformer-based models can ‘suffer from long training and inference times’.

## 3. Methodology

### 3.1. Data Collection

The Newcastle Urban Observatory (https://newcastle.urbanobservatory.ac.uk/, accessed on 10 January 2026), one of the largest public-facing urban sensing networks in the United Kingdom, provides real-time monitoring of various urban metrics across Newcastle upon Tyne. Each monitoring station integrates three key components: an IP-camera for video capture, a Raspberry Pi processing unit (Raspberry Pi Ltd., Cambridge, UK) enhanced with a Google Coral Edge TPU AI-accelerator (Google LLC, Mountain View, CA, USA), and a cellular modem for data transmission [23]. The system conducts real-time video analytics, performing object detection and tracking while recording line-crossing events. This processed data is then transmitted to a central backend system, which serves as our primary data source for subsequent analysis.

**Figure 2:**
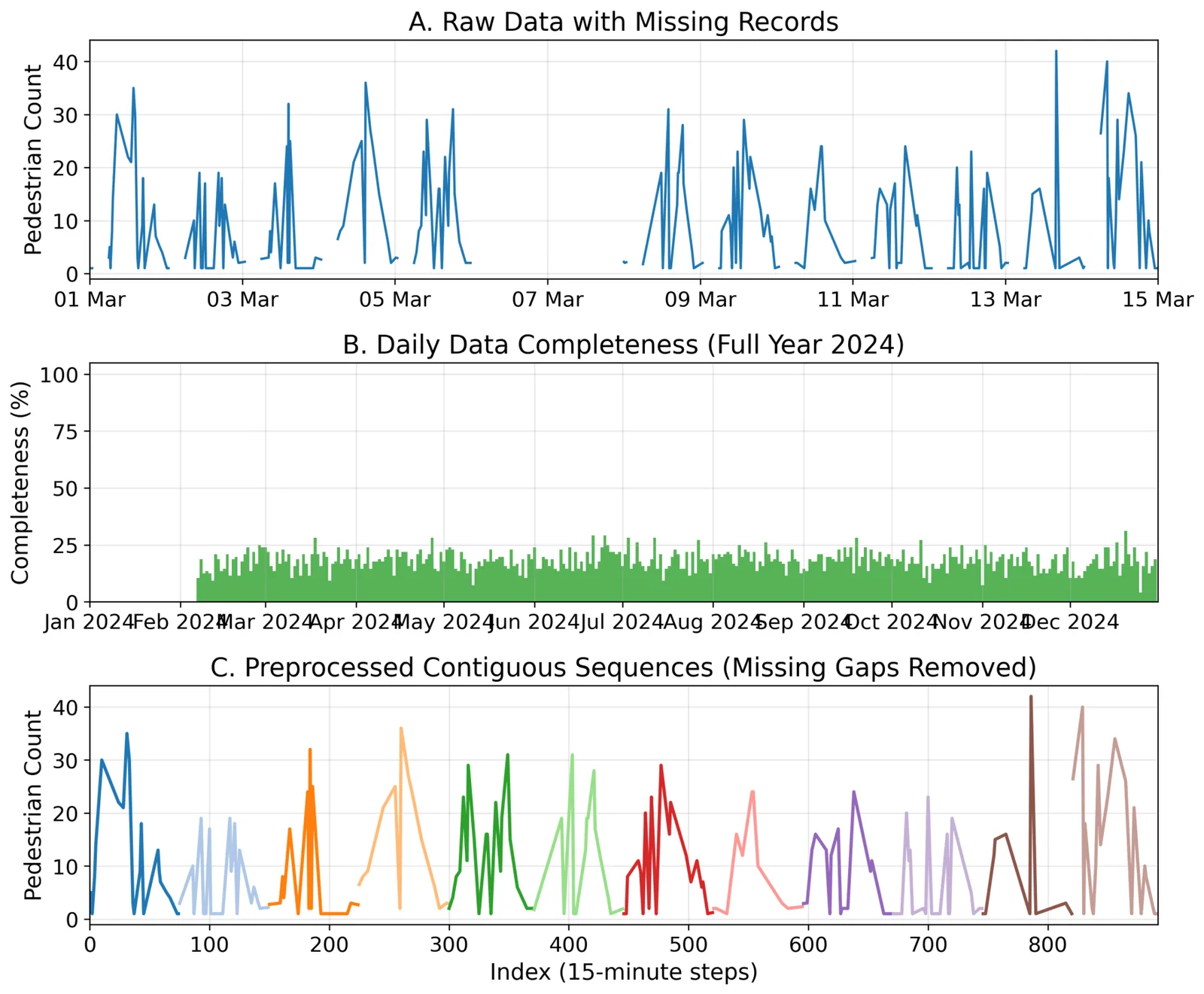
Raw data (**A**), percentage of records recorded per day (**B**), and preprocessed data for an example sensor (**C**), where distinct colours in (**C**) denote individual detected consecutive sequences without missing data.

The data from the pedestrian sensors is aggregated at 15 min intervals, with each record containing the number of pedestrians detected within a scene over that time period. Figure 2A shows the raw data from a single sensor over 18 days, highlighting two distinct patterns of missing data: regular night-time gaps (a design feature of some sensors) and extended periods of data sparsity due to technical issues such as sensor failure or data transmission problems. Figure 2B quantifies this data incompleteness; there should ideally be 96 records per day (4 per hour), but few days achieve complete coverage and some days have no records at all.

### 3.2. Data Preprocessing

The data preprocessing stage executes functions related to data cleaning (data removal, data interpolation, and data sequencing), which are necessary before any feature engineering takes place. While there are deep learning models that can handle missing data, these models tend to be more complex and tuning them can be challenging [24]. Another option is using complex imputation methods to fill in the missing data, but these are often costly and can affect the integrity of the data [24]. A particular pitfall for pedestrian data is the temptation to zero-fill overnight gaps: because many sensors are configured not to record when no pedestrians are detected, absent night-time records are easily mistaken for genuine zero counts. As we show in Section 4, such artificial zero-imputation inflates the apparent length of consecutive sequences and injects spurious patterns into the training data. For these reasons, missing records are deliberately preserved as missing (NaN) rather than imputed, and a consecutive sequence detection and labelling algorithm has been developed [25]. Consecutive sequences are sections of the time series that exist without any missing data. It works by scanning through a DataFrame row by row, building sequences where the time difference between consecutive rows equals the recording interval of the sensor (normally 15 min), when there is a gap in the timestamps, or when it checks if the current sequence meets the minimum length requirement (window_size + horizon, as detailed in Section 3.4). Valid sequences are stored and numbered sequentially.

Two key hyper-parameters define the window structure [26]:

1. window_size: Determines how many historical time steps are included in each window. This parameter can be tuned to optimise model performance.
2. horizon: Specifies how far into the future the model predicts. This parameter is not tuned and is set based on the desired prediction length.

Figure 2C shows the outputs of the data preprocessing stage for the same sensor as A and B where *n* is the temporal index of each observation and value is the number of pedestrians observed. Each new consecutive sequence is shown as a new colour. The sequences in this example capture similar segments of the daily cycle before a missing record appears. This means each sequence is less than 96 records long, which limits the length of training windows that can be used in the machine learning pipeline (for example, a window size of 24 with a horizon of 24 requires at least 48 consecutive time steps, otherwise the sequence will be discarded).

Applying this pipeline across the network yields 110 viable sensors (those with at least 500 training, 100 validation and 100 test windows). Their aggregate quality profile is summarised in Table 1. Critically, once artificial zero-filling is removed, the average consecutive sequence length is only 3.34 h (median 2.81 h), far shorter than would be inferred from a zero-imputed dataset. This short-sequence characteristic—a direct consequence of the genuinely daytime-only nature of pedestrian activity—is the central data quality challenge addressed in this paper.

**Figure 3:**
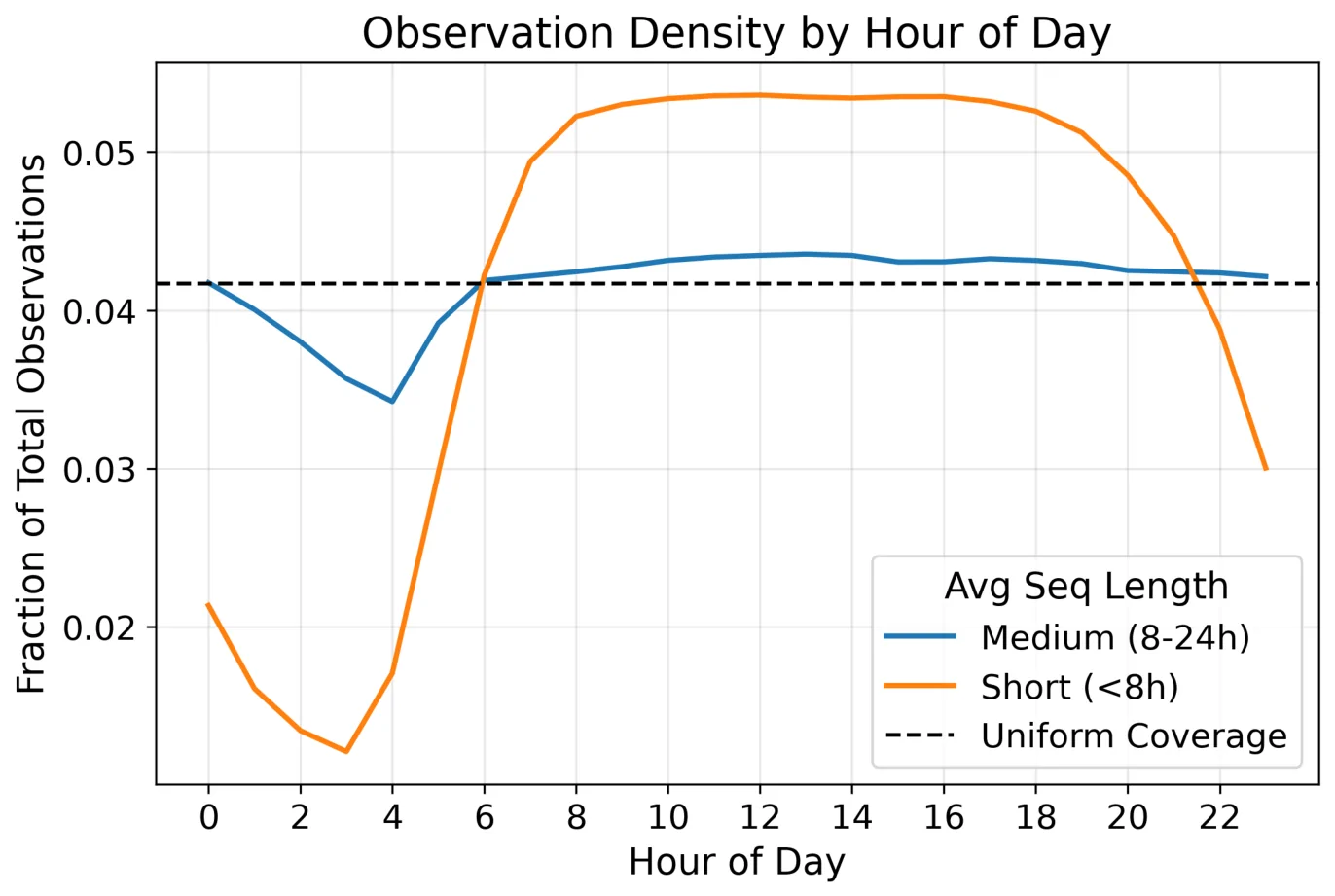
Fraction of sensors reporting an observation by hour of day. Short-sequence sensors (average sequence < 8 h) show a sharp overnight trough between 00:00 and 06:00, confirming their daytime-only operation.

The origin of these short sequences is made explicit by the network-wide observation density in Figure 3. Sensors with short average sequences (the majority) show a pronounced overnight trough, with the fraction of active sensors reporting falling to roughly 1% between 03:00 and 04:00 before recovering after 06:00. This diurnal signature confirms that the dominant source of missingness is not random sensor failure but the deactivation of sensors during low-activity hours, which repeatedly fragments the time series at daily boundaries.

### 3.3. Feature Engineering

The feature engineering stage tests the hypothesis that incorporating explicit temporal features alongside raw sensor data could improve prediction accuracy, particularly for longer horizons. This hypothesis proposes that combining harmonic decomposition through Lomb–Scargle periodograms [27,28] while deep learning would enable better capture of both cyclical patterns and irregular fluctuations in pedestrian movement. Crucially, because standard Fast Fourier Transforms cannot handle missing values, the Lomb–Scargle periodogram is computed directly on the raw, gapped time series for each sensor, avoiding the need for prior interpolation.

The proposed dual-representation strategy—using both raw time series and engineered temporal features—provides a more robust foundation for handling the inherent periodicities in urban mobility data while maintaining resilience to gaps in sensor observations. In total, 11 engineered temporal features are concatenated with the raw pedestrian count to form a 12-dimensional multivariate input vector. These features include the following:

- Harmonic features (eight variables): The sine and cosine components of four dominant frequencies identified via the Lomb–Scargle periodogram (half-day, daily, weekly, and quarterly/yearly).
- Timestamp encoding (three variables): Cyclical encodings of the time of day, day of week, and a binary weekend/holiday indicator.

To test this hypothesis, the methodology specifically differentiates between short-term (2 h) and longer-term (6 h) prediction horizons to evaluate how feature complexity requirements scale with prediction length.

The features are then normalised using the Standard Scaler equation $X_{i}^{\prime }=\frac{X_{i}-\mu }{\sigma }$, where $X_{i}$ is the original value, μ is the mean of the feature, and σ is the standard deviation of the feature. To prevent data leakage, these normalisation parameters (μ and σ) are fitted exclusively on the training data split for each sensor, and then applied to scale the validation and test sets. The standard scalar normalisation technique is chosen to ensure that any future data points falling outside of the existing range can be scaled appropriately, as a min–max scaler would not be appropriate in this case [29]. Calculating the mean and standard deviation in the scaling process also serves as a check for future data drift; if movement patterns change over time, the mean is likely to be non-stationary.

### 3.4. Data Loading

**Figure 4:**
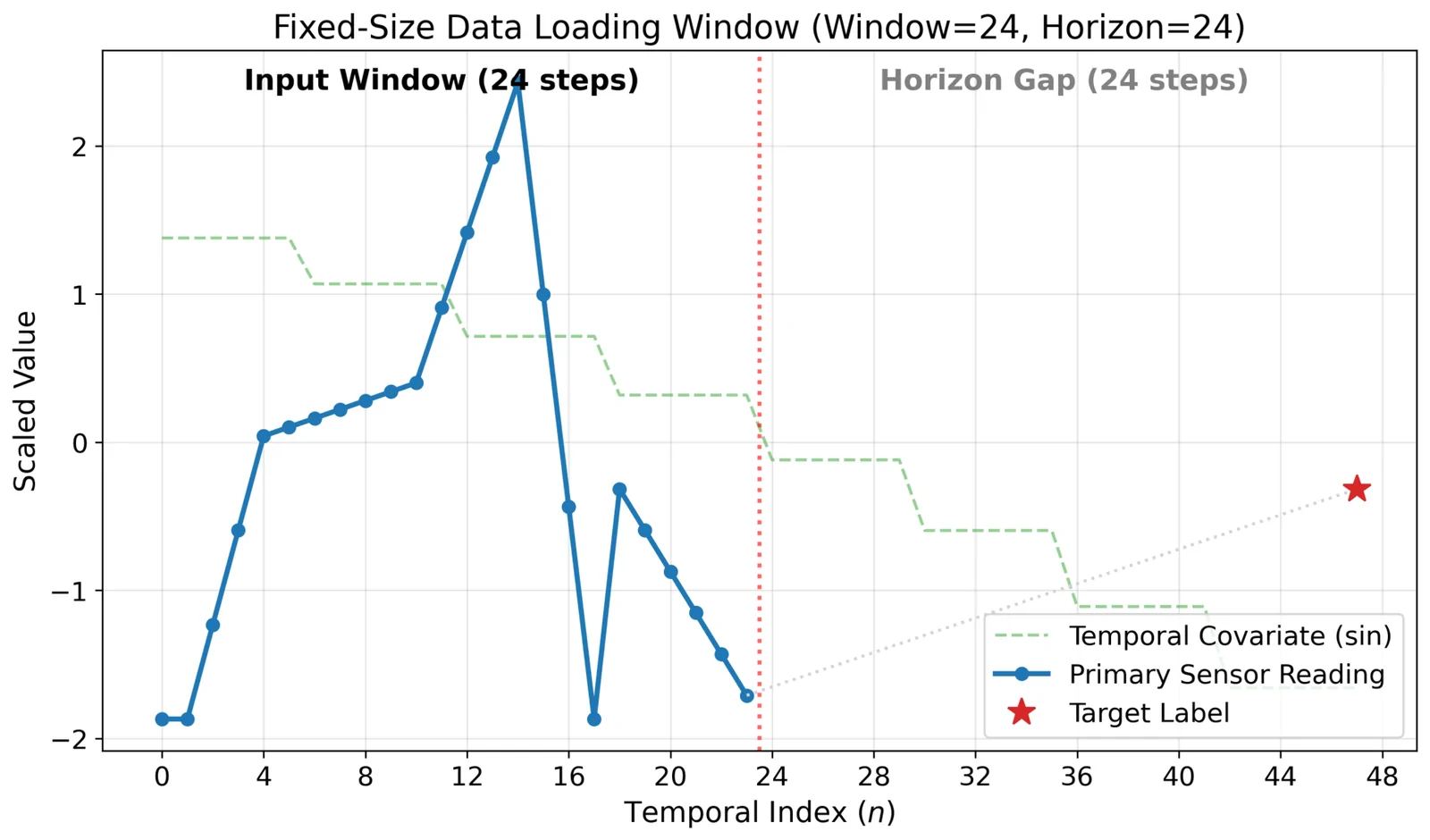
Example window used in the data loading process. The primary sensor sequence is shown in blue up to the end of the input window, the engineered temporal covariate is shown as a green dashed line across the sequence, and the target forecast point is indicated in red. The grey dashed line shows that the target label is the same variable as primary sensor reading.

For recurrent neural networks (such as LSTMs), a number of approaches to data loading can be used. A common approach for time-series data is using fixed-sized windows [30,31]. An example fixed-size window is shown in Figure 4 (*n* is the temporal index of each observation and value is the scaled pedestrian count). A fixed-size window contains multiple sequences of data: the primary sensor readings (shown in blue) which are only known up to the end of the input window, and an additional engineered signal covariate (e.g., a temporal feature, shown as a dashed line). This signal covariate is fully deterministic and thus is known in advance across the entire sequence, including the horizon gap and target point. Each window is associated with a label (shown in red) which represents a future data point that the model aims to predict.

In the example plot, a window size of 24 and horizon of 24 are used. This means the model is trained to predict pedestrian flow 6 h into the future using the previous 6 h of data. The prediction is made recursively, using the standardised count values and the additional temporal features from within each window.

### 3.5. Model Training

The evaluation was conducted across all 110 viable sensors in the network. For each sensor, the available chronological sequences are partitioned into training (approximately 70%), validation (15%), and test (15%) splits. A sensor was deemed viable if it yielded at least 500 training windows, 100 validation windows and 100 test windows at the minimum sequence length (window_size + horizon); 110 of the network’s pedestrian sensors met this criterion.

Eight forecasting models were compared at two horizons—h=8 (2 h) and h=24 (6 h)—comprising multivariate and univariate variants of both an LSTM and a Transformer encoder (Trans-MV/Trans-UV), weekly and daily seasonal naïve baselines, a persistence baseline, and ARIMA. The multivariate/univariate comparison assesses whether incorporating engineered temporal and frequency features enhances prediction accuracy, while the architectural comparison (LSTM vs. Transformer) evaluates whether self-attention mitigates the sequence fragmentation constraint.

A separate interpolation experiment examined whether gap-filling could overcome the short-sequence problem. Five strategies were evaluated: no interpolation (baseline), night-time zero-fill (00:00–06:00), linear interpolation, cubic spline interpolation, and *k*-nearest-neighbours (KNN) interpolation. This expands substantially on the original night-zero-only experiment and allows the genuine value of principled imputation to be separated from the artefacts of naive zero-filling. To keep comparisons fair across strategies, error metrics for the interpolated variants are reported on the originally observed (real) time steps only.

Rather than re-tuning hyperparameters, we leveraged the optimal hyperparameters established in prior experiments on a small high data completeness subset of the sensors. In that previous phase, models were systematically tuned through an optimisation process consisting of 100 trials per experimental condition. The original hyper-parameter search space is presented in Table 2, including the window size (ranging from 4 to 48 time steps), batch size (32 to 128), learning rate ($10^{-5}$ to $10^{-1}$), scheduler step size (3 to 7 steps), model architecture (LSTM or GRU), hidden dimensions (32 to 256 units), number of layers (1 to 3), and dropout rate (0 to 0.5). By holding these previously tuned architectures constant in this study, we explicitly test the models’ ability to generalise to the much broader, noisier distribution of data quality found across the entire network. While this approach risks favouring high-quality sensors, it serves as a rigorous stress test: it establishes an upper bound of model capability on ideal data, allowing us to isolate exactly how and why performance degrades when this ideally tuned model is exposed to the severe sequence fragmentation typical of real-world deployments.

The original tuning process utilised the Optuna library [32] in conjunction with MLflow [33] for experiment tracking, with RMSE (root mean square error) selected as the optimisation metric, following established practices in time-series prediction tasks [34].

### 3.6. Model Evaluation

To evaluate the model, a range of metrics are used. The primary metric (used for hyper-parameter tuning) is the root mean squared error (RMSE). RMSE measures the difference between the predicted and actual values and is more sensitive to outliers than the mean absolute error (MAE). Mean absolute percentage error (MAPE) is also used as it can show the relative error between predicted and actual values. To enable fair comparison across sensors with very different pedestrian volumes, we also report the coefficient of variation of the RMSE (CV-RMSE), which normalises RMSE by the mean observed count. Because per-sensor error distributions are highly skewed, aggregate results are reported as medians across the 110 sensors.

To establish that observed differences between models are statistically meaningful rather than artefacts of the aggregate median, pairwise Diebold–Mariano (DM) tests [35] are computed per sensor, and the fraction of sensors on which a model is significantly better (p<0.05) is reported. Relationships between data quality metrics and forecast error are quantified using Spearman rank correlations (with false-discovery-rate correction) and partial correlations and are summarised with a random forest meta-model that predicts per-sensor error from quality features.

Sensor data quality is characterised using four primary metrics: average sequence length, the standard deviation of sequence length, sequence count, and data completeness. Average sequence length, defined as the mean length of consecutive record sequences, provides insight into data continuity; its standard deviation and the total sequence count capture how the record stream is fragmented. Data completeness is calculated as the ratio of actual records to expected records during active hours. A diurnal capture ratio—the fraction of spectral power at the 24 h period—additionally quantifies how strongly each sensor expresses the daily activity cycle.

### 3.7. Hyper-Parameter Tuning

The hyper-parameters established for the deep learning models are detailed in Table 3. The tuned architectures are consistent across univariate and multivariate variants, with all employing 64 hidden dimensions, a batch size of 128, and a dropout rate of 0.1. The primary architectural distinction lies in the depth of the recurrent networks: the multivariate LSTM requires two layers to process the complex interactions between raw counts and the 11 engineered temporal features, whereas the univariate LSTM performs best with only a single layer. (The Transformer models uniformly use two encoder layers.) The multivariate models also employ a slightly more conservative learning rate (0.0005 versus 0.001). Importantly, these unified architectures were held constant when evaluating performance across both the short (h=8) and long (h=24) forecasting horizons, ensuring that any performance variations are strictly attributable to the forecasting horizon and input features rather than model-specific tuning artifacts.

## 4. Results

### 4.1. Model Comparison

Table 4 reports median forecast error across all 110 sensors for the models with no interpolation applied. The multivariate models (LSTM-MV and Trans-MV) demonstrate robust performance and emerge as the strongest models at both horizons, performing nearly identically (e.g., median RMSE of 6.19 vs. 6.13 at h=8). At h=24 they achieve an RMSE of 4.75 and 4.83, respectively, which is approximately 19% lower than their univariate counterparts. The advantage of the deep learning models over the seasonal naïve and persistence baselines is large, and persistence degrades severely at the longer horizon (CV-RMSE 186%). ARIMA is only viable on 12 of the 110 sensors: its requirement for 192 consecutive steps (48 h) of continuous data excludes the vast majority of sensors, whose average sequence length is approximately three hours, and where it does fit it is not competitive. This scaling behaviour is itself an important quality-related finding: classical models that assume long, uninterrupted histories are simply inapplicable to the bulk of a real pedestrian sensor network.

To confirm that the deep learning advantage reflects a genuine per-sensor improvement rather than a handful of influential sensors dominating the median, pairwise Diebold–Mariano tests were computed for every sensor (Table 5). The LSTM-MV is directionally better than every baseline on essentially all sensors, and this superiority is individually significant (p<0.05) on 92–98% of sensors at h=8 and on 79–99% at h=24. Even the narrowest margin—against the weekly seasonal naïve at h=24—remains significant on 79% of sensors. When comparing LSTM-MV to Trans-MV, the DM test shows no significant difference on the vast majority of sensors (significant on only 13.6–25.5%), confirming the two architectures are statistically equivalent. This is far stronger evidence than an aggregate comparison alone and rules out the possibility that the median advantage is driven by outliers.

The value of the engineered temporal features is isolated by a feature-ablation study (Table 6), in which each harmonic group is removed in turn from the LSTM-MV. The single most impactful feature is the half-day harmonic: removing it increases RMSE by 1.4% at h=8 (6.16 to 6.25) and by 5.1% at h=24 (4.69 to 4.93). This is consistent with pedestrian flow exhibiting strong 12 h periodicity through morning and evening commute peaks. The daily harmonic contributes comparatively little on its own, most likely because the half-day component already captures the dominant diurnal structure. That the multivariate features matter more at the longer horizon explains why the LSTM-MV advantage is sustained as the forecast horizon grows.

### 4.2. The Quality–Performance Relationship

Having established that the LSTM-MV is the strongest model, we now examine which data quality metric best explains why some sensors are far easier to forecast than others. Table 7 reports Spearman rank correlations between four quality metrics and LSTM-MV RMSE, together with partial correlations that control for confounding. All four raw correlations are significant after false-discovery-rate correction. Sequence-based metrics—average sequence length and its standard deviation—are the strongest correlates (ρ≈0.72–0.74), while completeness is only moderately correlated (ρ≈0.48) and sequence count is negatively correlated (ρ≈−0.53; more fragments imply shorter, less useful sequences).

These partial correlations isolate the true driver of model performance. After controlling for completeness, average sequence length retains a strong partial correlation with error (ρ=0.617–0.650, $p<10^{-12}$). Conversely, after controlling for sequence length, completeness becomes non-significant (ρ=−0.169, p=0.078 at h=8). Completeness therefore appears predictive only because it co-varies with sequence length; it carries little independent information. In other words, it is the structure of missing data—how it fragments the record stream into short sequences—that governs forecastability, not the amount of missing data.

To test whether this fragmentation sensitivity is architecture-specific, we trained equivalent multivariate Transformer encoder models with identical hyper-parameter budgets. As established in Table 4, the Transformer models achieve statistically indistinguishable accuracy from the LSTMs. Critically, the Spearman correlation between sequence length and forecast error is identical across architectures (e.g., ρ=0.714 for Trans-MV at h=8, compared to 0.715 for LSTM-MV). Segmented regression breakpoints similarly cluster around 2 to 3 h for all models. This confirms that the quality-awareness diagnostic generalises across model families: the framework identifies data-level constraints, rather than limitations of any specific deep learning architecture.

#### A Three-Hour Sequence-Length Threshold

**Figure 5:**
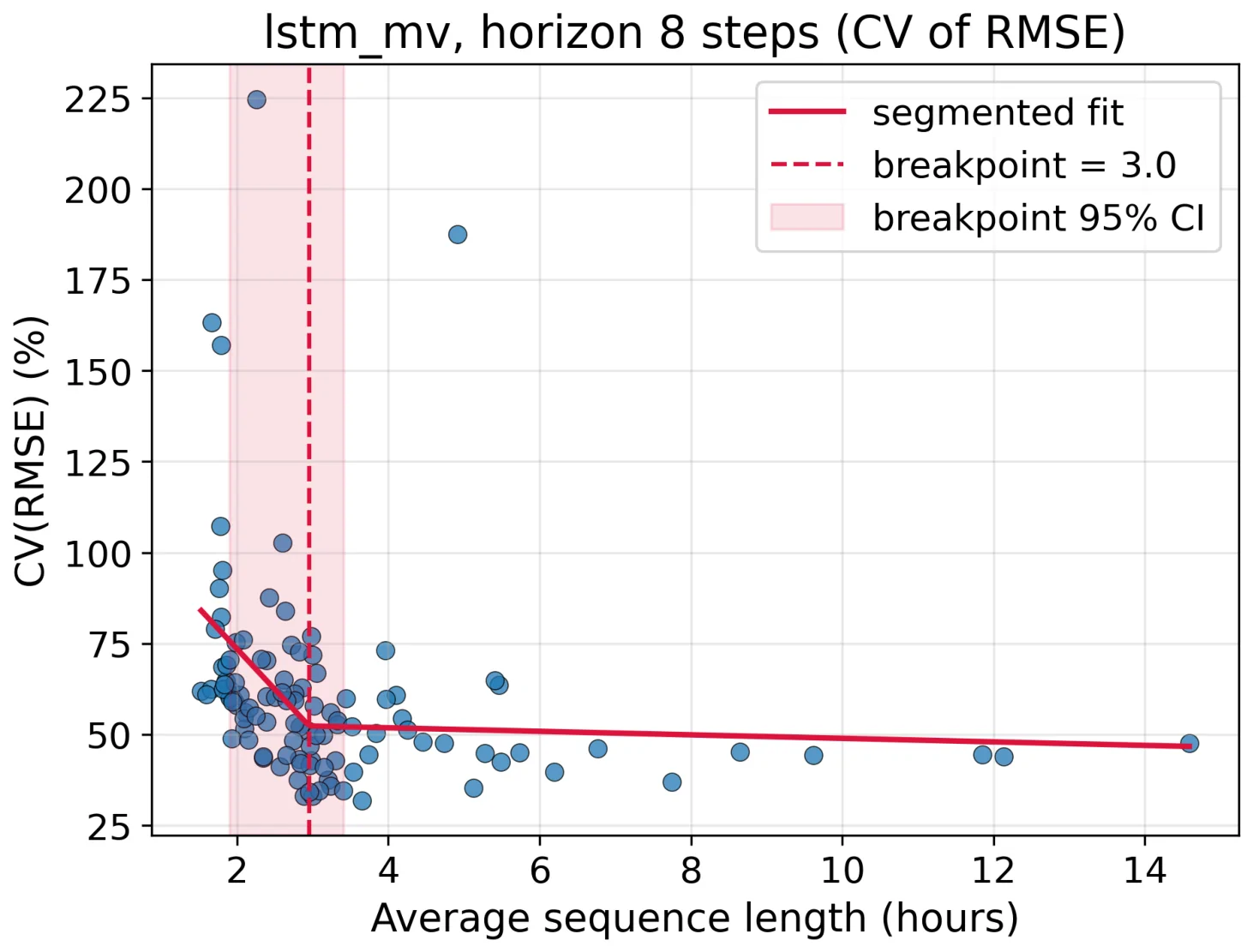
Segmented regression of CV-RMSE against average sequence length for the LSTM-MV at h=8. Circles represent individual sensors, and the solid line shows the fitted segmented regression with a breakpoint at approximately three hours separating a steep decline phase from a plateau.

The dependence of relative error on sequence length is not linear. Fitting a piecewise-linear (segmented) regression of CV-RMSE against average sequence length reveals a clear breakpoint (Table 8, Figure 5). At h=8 the breakpoint is 3.0 h (95% CI [1.9,3.4]): below it, CV-RMSE falls steeply at approximately −22% per additional hour of sequence length, whereas above it the slope flattens to about −0.5% per hour. The segmented model is favoured over a simple linear fit (ΔAIC =+7.2). The h=24 breakpoint is similar (2.6 h) though less sharply supported (ΔAIC =+0.4). This three-hour threshold corresponds to roughly twelve consecutive 15 min records—about half a diurnal cycle—suggesting that the LSTM needs at least half a day of uninterrupted context before it can learn useful temporal structure. Below this point, additional records do little to help; above it, the network already has enough context and further continuity yields diminishing returns.

### 4.3. Interpolation Experiment

Having quantified how sequence fragmentation harms performance, we ask whether gap-filling can recover the lost continuity. Five strategies were applied to the LSTM-MV and evaluated on the originally observed time steps only (Table 9). By isolating genuine missing data from active periods, we can evaluate the true efficacy of these methods in overcoming temporal gaps.

Three findings emerge. First, principled gap-filling genuinely helps at the short horizon: linear interpolation reduces median RMSE by 17% at h=8 (6.16 to 5.10), and spline interpolation gives a similar improvement. Second, naive night-time zero-fill remains ineffective and is actively harmful at the longer horizon: it improves RMSE by only 1.3% at h=8 but increases RMSE by 24% at h=24 (4.69 to 5.83), because injecting artificial zeros during overnight hours creates spurious patterns that mislead the model over the longer windows it must span. Third, the choice of strategy interacts with the choice of metric: the no-interpolation baseline retains the best RMSE and CV-RMSE at h=24, whereas linear interpolation achieves the best MAPE (65.6%). This trade-off arises because MAPE penalises relative errors on low-count windows most heavily, and interpolation smooths precisely those low-count periods. Practitioners should therefore choose an interpolation strategy according to the error metric that matters for their application. Critically, the failure of night-time zero-filling highlights a hazard specific to event-triggered sensors: when missing data arises from hardware configuration (e.g., deactivating during idle periods) rather than confirmed zero events, naive zero-filling injects spurious patterns that degrade longer-horizon forecasts.

### 4.4. Predictive Modelling of Performance

The strong, structured relationship between quality metrics and error suggests that per-sensor performance can be predicted before a model is trained—valuable guidance for network operators deciding whether their data are fit for forecasting. A random forest regressor [36,37] was trained to predict per-sensor RMSE from four quality features: average sequence length, its standard deviation, sequence count, and completeness. The model achieves an out-of-bag $R^{2}$ of 0.81 at h=8 and 0.80 at h=24 (Table 10), demonstrating strong predictive capability across the 110 sensors.

**Figure 6:**
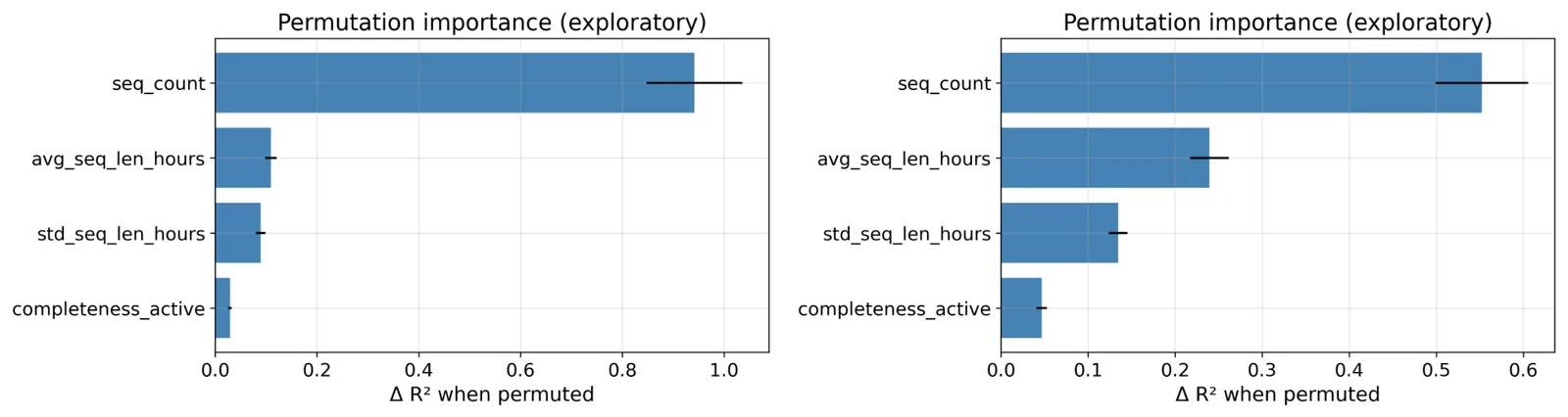
Random forest permutation importance for predicting per-sensor RMSE at h=8 (**left**) and h=24 (**right**). Sequence count dominates.

Permutation importance (Figure 6) identifies sequence count as the dominant predictor, far ahead of the other features at h=8. This is consistent with the mechanism established above: sensors with more sequences have, on average, shorter sequences, and it is short sequences that drive error. Completeness is the least important feature, reinforcing that it carries little independent predictive information.

**Figure 7:**
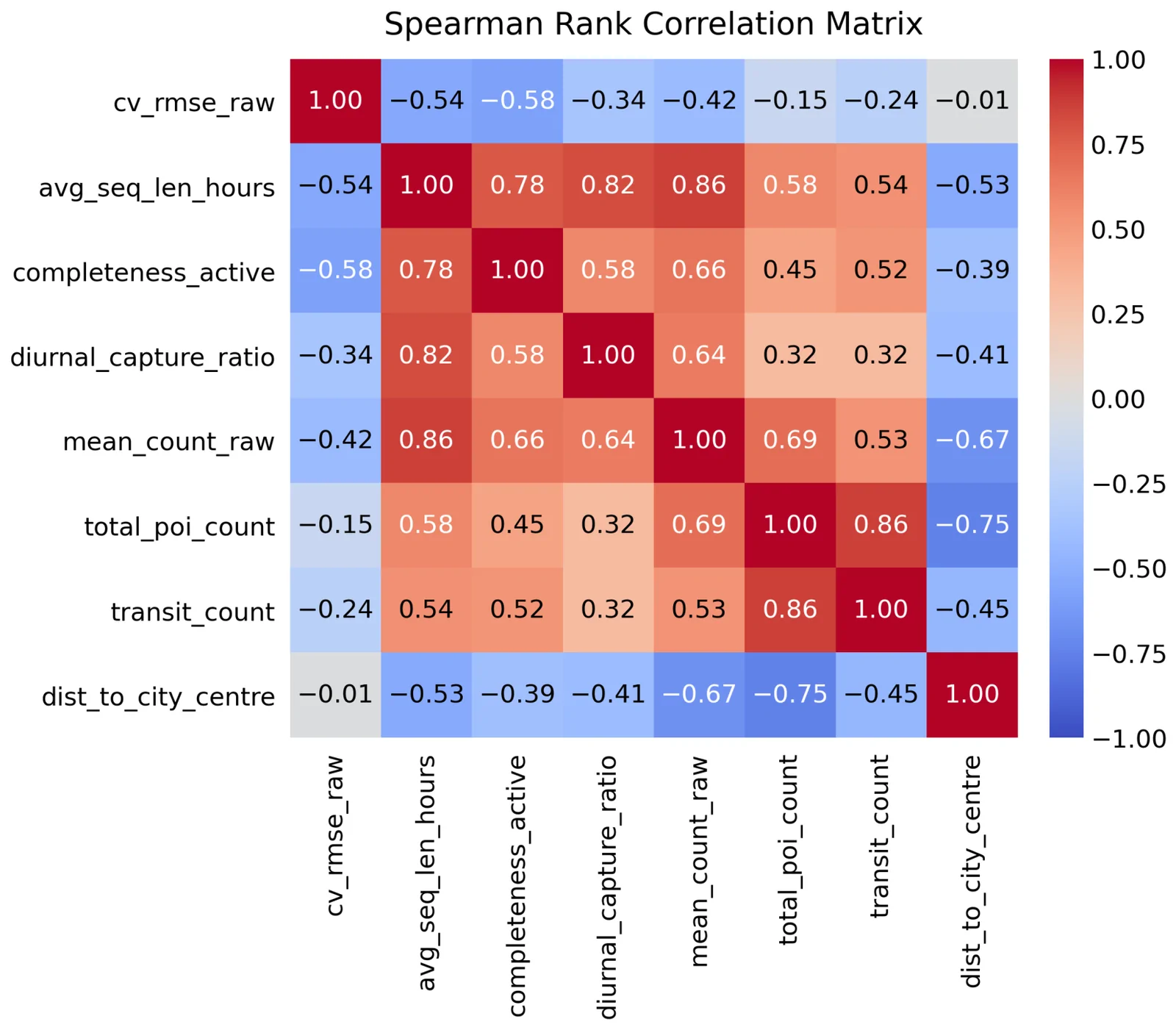
Correlation matrix of quality, volume and spatial metrics across the 110 sensors.

To confirm that fragmentation penalises the model because it shortens sequences—rather than merely because short sequences happen to occur on low-volume sensors—an ordinary least squares regression of CV-RMSE on average sequence length, mean pedestrian count, and their interaction was fitted. Average sequence length remained a significant predictor (p=0.038), whereas neither pedestrian volume (p=0.403) nor the interaction term (p=0.182) was significant. Fragmentation therefore harms performance through its effect on sequence length per se, independent of how busy a sensor is. The full set of pairwise relationships between quality, volume and spatial metrics is shown in Figure 7.

### 4.5. Spatial and Contextual Analysis

**Figure 8:**
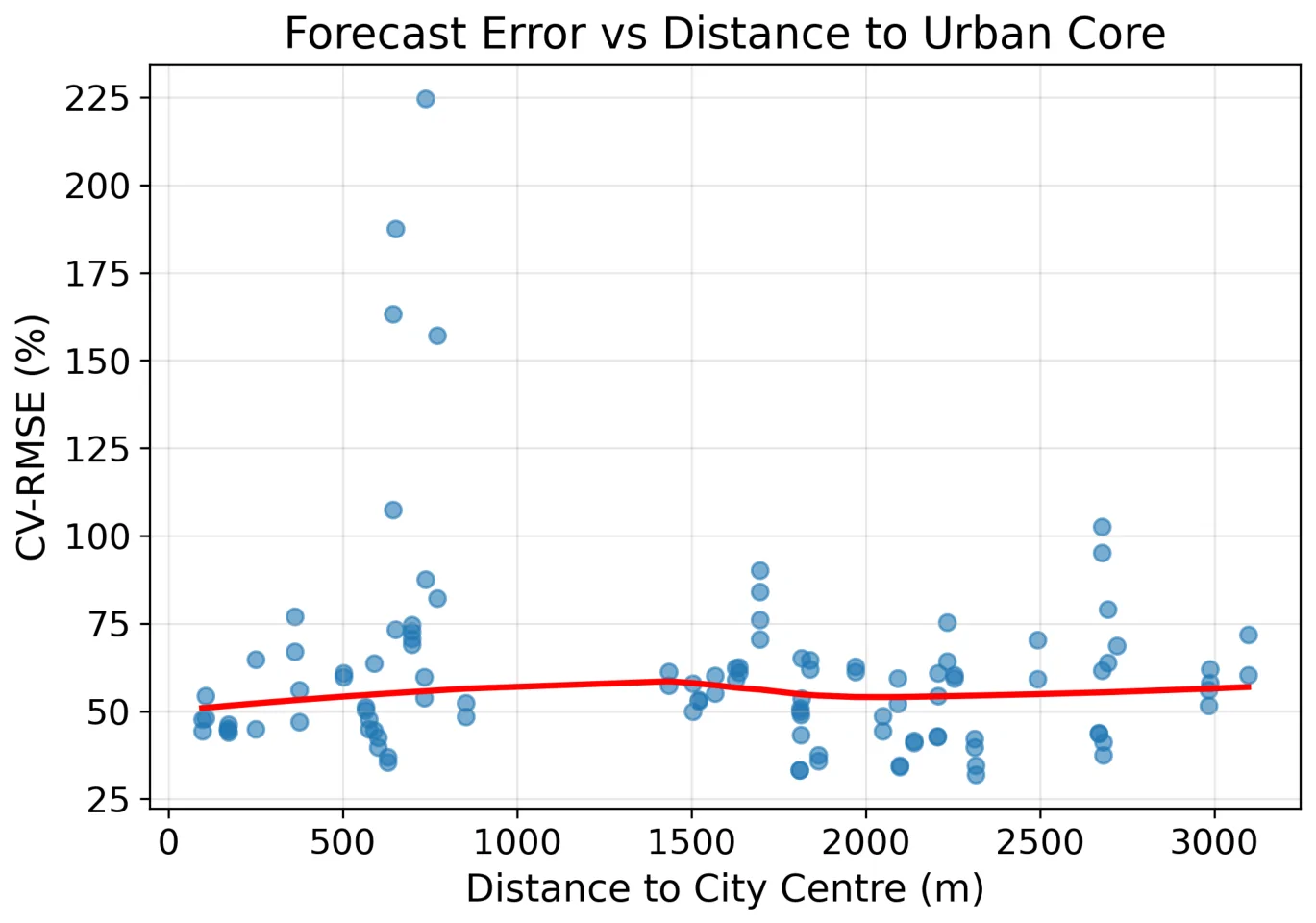
Forecast error (CV-RMSE) against distance to the city centre. Individual points represent sensors, and the line shows the fitted trend line indicating that error increases with distance from the urban core.

The preceding analysis establishes what makes a sensor forecastable; this section asks where such sensors are, linking data quality to the built environment. Forecast error is not spatially random: CV-RMSE tends to decay with distance from the city centre (Figure 8), indicating that a sensor’s location carries information about its data quality.

To test the role of the built environment directly, sensors were stratified by transit density and their diurnal capture ratio compared. A Mann–Whitney *U* test found that sensors in high-transit locations have significantly stronger diurnal signals than those in low-transit locations (p=0.0026). Transit-rich environments thus generate the regular, strongly periodic pedestrian flows that the LSTM can most readily learn.

**Figure 9:**
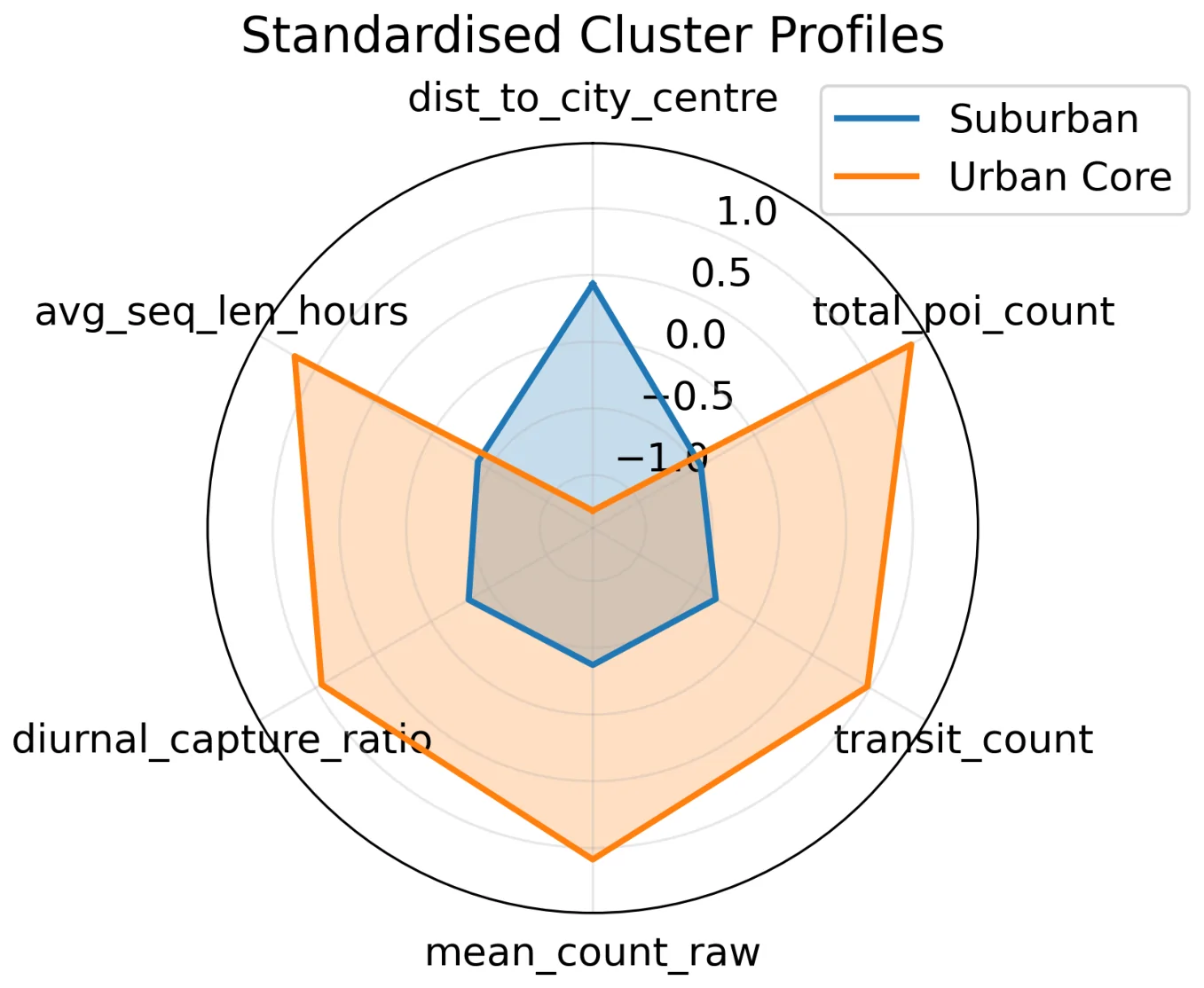
Radar chart contrasting the urban-core and suburban sensor clusters across quality, volume and built-environment dimensions.

Silhouette-optimal *k*-means clustering on combined quality and geospatial features identifies two natural sensor groups (Table 11, Figure 9). The urban-core cluster (28 sensors) sits close to the centre amid dense points of interest and transit, with high pedestrian volumes, long sequences and strong diurnal capture. The suburban cluster (82 sensors) is spatially distant, with fewer amenities, shorter sequences and weaker periodicity. The urban-core cluster has markedly lower relative error (median CV-RMSE 47.7% versus 59.3%); a Kruskal–Wallis test on CV-RMSE between the clusters is marginal (p=0.067), but the sizeable difference in medians indicates that locational profile is a meaningful determinant of forecasting difficulty. Notably, the urban-core sensors have a much higher absolute RMSE, reflecting their far larger pedestrian counts; it is the normalised (relative) error that reveals their superior predictability.

**Figure 10:**
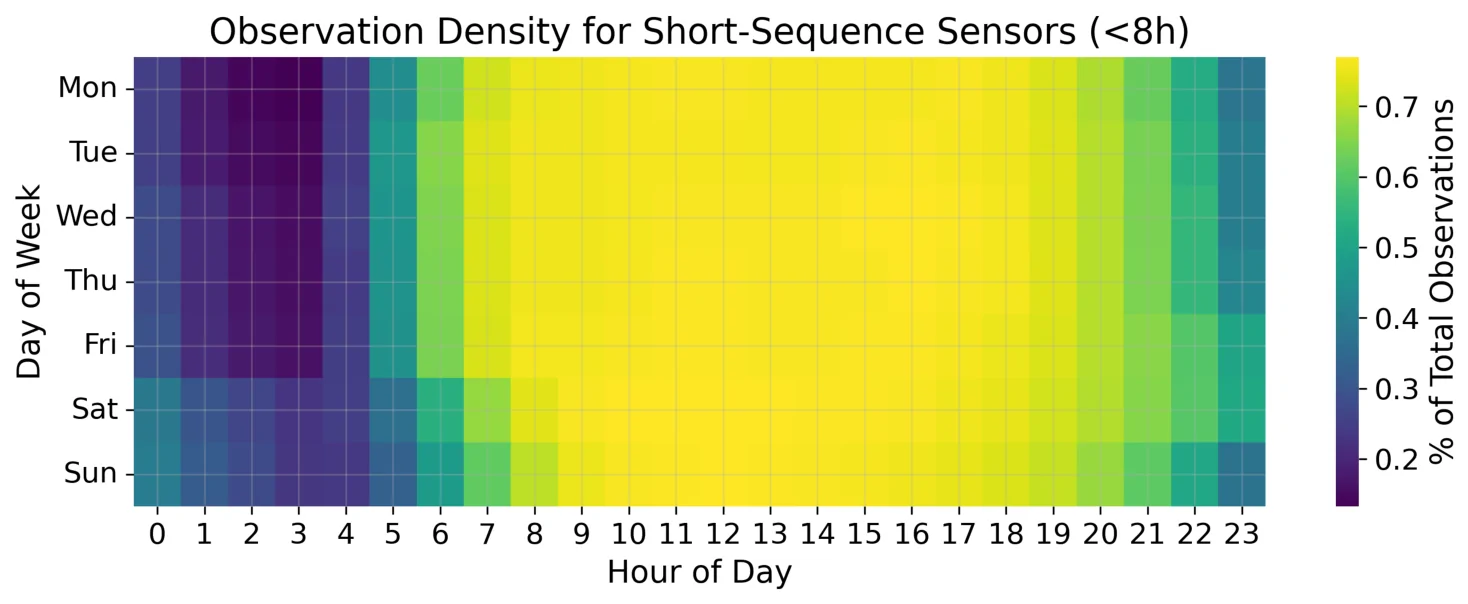
Observation-density heatmap (day of week versus hour of day) for short-sequence sensors, showing the dominant overnight gap.

Finally, the two-dimensional missingness map in Figure 10 (day of week versus hour of day, for short-sequence sensors) shows that the fragmentation is overwhelmingly driven by the overnight deactivation pattern rather than by weekday/weekend effects, explaining why sequence lengths so rarely exceed the three-hour threshold identified above.

## 5. Discussion

### 5.1. Data Quality Assessment and Implications

The investigation into IoT pedestrian data streams reveals that data quality challenges stem from multiple factors, aligning with [7]’s comprehensive analysis of AIoT data quality issues. While their work established a broad framework for understanding AIoT data quality, our findings specifically demonstrate the critical importance of temporal continuity for predictive-modelling performance and quantify it. Analysing 110 sensors on a dataset in which overnight gaps are preserved rather than zero-filled, we find that the length of consecutive data sequences—not overall completeness—is the dominant quality dimension. This extends traditional data quality taxonomies by foregrounding the temporal structure of missing data in urban sensing applications.

Crucially, this analysis decouples the effects of overall missing data volume and temporal fragmentation. Partial-correlation analysis shows that completeness only appears predictive because it co-varies with sequence length: once sequence length is controlled for, completeness has no independent association with error (ρ=−0.169, p=0.078), whereas sequence length remains strongly predictive after controlling for completeness (ρ=0.617–0.650, $p<10^{-12}$). Any apparent negative effect of high completeness is therefore a confound, not a genuine phenomenon, and—as our OLS (ordinary least squares) interaction test confirms—fragmentation harms the model by cutting sequences short rather than because it happens to occur on low-volume sensors. Interpreting this through [8]’s work on temporal dependencies, the inability to observe a sufficient span of the diurnal cycle is what impairs the model.

Our segmented-regression analysis translates this into a concrete, actionable requirement for the Newcastle network. The breakpoint is approximately three hours of average sequence length, roughly half a diurnal cycle and about twelve consecutive 15 min records. Below this threshold, relative error deteriorates rapidly (−22% per hour); above it, additional continuity yields diminishing returns (−0.5% per hour). We do not present this three-hour figure as a universal standard. Instead, we present the underlying data quality assessment framework as a methodological tool for operators in other cities. While our results do not guarantee that an identical breakpoint—or indeed any single breakpoint—will emerge in different urban contexts, they strongly suggest that such structural constraints plausibly exist elsewhere. Applying this analytical framework empowers network operators to discover the specific sequence-length thresholds governing their own infrastructure, ultimately helping them understand the true nature and predictive utility of their sensor data.

The comparison of univariate and multivariate models is likewise clarified at scale. Across 110 sensors the multivariate LSTM is the strongest model at both horizons, and Diebold–Mariano tests show this advantage is significant on 79–98% of individual sensors rather than merely in aggregate. Feature ablation attributes much of this to the half-day harmonic, consistent with [38]’s emphasis on feature engineering for time-series analysis; the growing contribution of the temporal features at the longer horizon indicates they help compensate for limited context when forecasting further ahead.

Principled gap-filling shows clear, metric-dependent value: linear interpolation reduces RMSE by 17% at the 2 h horizon. However, ref. [24]’s caution remains apt for naive schemes—night-time zero-filling is ineffective at the short horizon and actively harmful at the longer one, since injected zeros create spurious patterns. The practical recommendation for event-triggered networks is to prefer simple, faithful interpolation (e.g., linear) where the target metric rewards it and to avoid zero-imputing gaps that actually represent sensor inactivity rather than confirmed zero traffic. Ultimately, these findings underscore the value of developing predictive models that do not strictly require continuous inputs. While principled interpolation can yield improvements at short horizons, any form of data imputation risks introducing artifacts—such as the spurious overnight patterns observed here—that actively mislead models over longer windows. Forecasting architectures capable of natively handling irregular sampling or explicit missingness (such as time-aware attention mechanisms or specialized RNNs) would avoid the need to hallucinate data entirely, preserving the fidelity of the raw pedestrian signal while naturally bridging temporal gaps.

Finally, the spatial analysis links these statistical findings to the physical city. Forecast error decays with proximity to the centre, transit density is significantly associated with stronger diurnal signals (p=0.0026), and clustering cleanly separates a predictable, transit-rich urban core from a less predictable suburban periphery. Observable built-environment attributes—points of interest, transit provision, distance to centre—can thus serve as low-cost proxies for expected data quality, connecting machine learning performance to urban-planning context.

### 5.2. Implementation, Limitations, and Practical Recommendations

The findings from this research have led to specific modifications in data collection protocols for the Urban Observatory’s pedestrian sensors, particularly the implementation of completeness metadata and sensor heartbeat monitoring. Sensor heartbeat monitoring outputs a boolean value that allows us to differentiate between periods where the sensor is not outputting data because there is no activity and when the sensor is not working—precisely the distinction whose neglect motivates zero-imputation and corrupts sequence statistics. These changes align with [17]’s recommendations for enhanced data provenance in IoT systems, and the heartbeat mechanism in particular operationalises [39]’s proactive data quality management principles. Our quality-aware pipeline demonstrates the feasibility of automated quality assessment in real-time sensor networks, addressing a key challenge identified by [9].

Several limitations remain. The rapid drop-off in data availability beyond a 6 h horizon, stemming from the fixed-window training approach and the overnight gaps, still constrains investigation of longer horizons; sequence lengths in this network are simply too short to support them under the current windowing methodology. Optimising hyper-parameters for average performance across all sensors, while efficient, forgoes sensor-specific tuning that might benefit atypical sensors. The random forest meta-model, although exhibiting strong overall reliability (out-of-bag $R^{2}=0.81$ on 110 sensors), still exhibits wide cross-validation spread on the hardest sensors, and its predictions should be treated as guidance rather than guarantees. While this study is based on a large network of 110 sensors, validation on other cities remains necessary.

## 6. Conclusions

This study revealed several insights about the relationship between data quality and predictive-modelling performance in urban sensor networks. Working across 110 sensors, it demonstrates that the temporal structure of missing data—captured by average sequence length—governs forecast accuracy, with a clear requirement of approximately three hours (half a diurnal cycle) of consecutive data; overall completeness, once confounding is removed, has no independent predictive power. The multivariate LSTM is the best forecaster at both horizons, significantly so on the large majority of individual sensors, and principled interpolation (linear) can further reduce short-horizon error whereas naively zero-filling periods of sensor inactivity proves actively harmful. Basic data quality metrics predict per-sensor performance well enough ($R^{2}=0.81$) to serve as a pre-deployment screening tool, and observable built-environment attributes act as spatial proxies for that quality.

Future research should focus on several avenues. Sensor-specific optimisation strategies would allow more nuanced tuning across diverse sensor configurations. Methods that exploit spatial dependencies between sensors—motivated directly by the clustering and distance-to-centre findings reported here—could enhance accuracy by borrowing strength across nearby locations. Integration of anomaly-detection algorithms incorporating multiple quality dimensions would enable more robust identification of sensor malfunctions, improving training-data quality. Finally, validation across additional cities and diverse urban environments would strengthen the generalisability of the three-hour threshold and the built-environment proxies, supporting the scaling of quality-aware predictive systems for broader smart-city applications.

## Figures and Tables

**Table 1:** Data quality profile across the 110 viable sensors (overnight gaps preserved as missing).

Quality Metric	Mean	Median	Std	Min	Max
Completeness (active hours)	0.743	0.756	0.121	0.364	0.952
Avg. sequence length (hours)	3.34	2.81	2.15	1.53	14.59
Std. sequence length (hours)	5.89	5.39	3.13	2.60	24.54
Sequence count	5089	5793	2636	404	9742
Mean raw count (ped./15 min)	22.70	12.41	30.23	4.25	178.25
Diurnal capture ratio	0.153	0.123	0.097	0.064	0.607

**Table 2:** Hyper-parameter tuning configuration.

Parameter	Suggestion Type	Value Range/Options
window_size	suggest_categorical	[4, 8, 12, 16, 24, 32, 48]
batch_size	suggest_categorical	[32, 64, 128]
lr	suggest_loguniform	$1\times 10^{-5}$ to $1\times 10^{-1}$
scheduler_step_size	suggest_int	3–7
model_type	suggest_categorical	[“lstm”, “gru”]
hidden_dim	suggest_categorical	[32, 64, 128, 256]
num_layers	suggest_int	1 to 3
dropout	suggest_uniform	0.0 to 0.5

**Table 3:** Hyper-parameters adopted for the deep learning models. Architectures share identical window size, batch size, dropout, and learning rate schedules. Models were evaluated at horizons h=8 and h=24 using these constant architectures.

Parameter	LSTM-UV	LSTM-MV	Trans-UV	Trans-MV
window_size	24	24	24	24
batch_size	128	128	128	128
hidden_dim/d_model	64	64	64	64
num_layers	1	2	2	2
nhead	–	–	4	4
dim_feedforward	–	–	128	128
dropout	0.1	0.1	0.1	0.1
learning_rate	0.001	0.0005	0.001	0.0005
scheduler_step_size	5	5	5	5
scheduler_gamma	0.7	0.7	0.7	0.7

**Table 4:** Median forecast error across 110 sensors (no interpolation). Best value in each column in bold. ARIMA is viable on only 12 sensors.

Model	n	h=8 (2 h)			h=24 (6 h)		
		RMSE	MAPE	CV-RMSE	RMSE	MAPE	CV-RMSE
LSTM-MV	110	6.19	69.2%	**56.0%**	**4.75**	80.8%	**62.1%**
Trans-MV	110	**6.13**	**68.9%**	56.2%	4.83	**80.2%**	62.5%
LSTM-UV	110	7.71	85.6%	62.4%	5.79	96.1%	68.9%
Trans-UV	110	**7.62**	**84.3%**	**61.8%**	**5.51**	**91.2%**	**66.8%**
Seasonal naïve (weekly)	110	8.14	83.9%	72.3%	7.62	119.8%	97.3%
Seasonal naïve (daily)	110	8.77	87.6%	77.3%	7.55	116.4%	94.9%
Persistence	110	12.01	125.9%	92.7%	16.88	366.2%	186.0%
ARIMA	12	64.82	312.1%	76.3%	75.44	537.7%	141.3%

**Table 5:** Diebold–Mariano tests, LSTM-MV versus each baseline. A negative median DM statistic indicates the LSTM-MV has the lower loss. “% Sig.” is the fraction of sensors with p<0.05.

Comparison	h	n	% Better	% Sig.	Median DM
LSTM-MV vs. Persistence	8	110	100%	98.2%	−9.56
LSTM-MV vs. S-naïve (daily)	8	110	100%	94.5%	−6.07
LSTM-MV vs. S-naïve (weekly)	8	110	99.1%	91.8%	−5.70
LSTM-MV vs. ARIMA	8	12	100%	100%	−9.68
LSTM-MV vs. Persistence	24	110	100%	99.1%	−8.60
LSTM-MV vs. S-naïve (daily)	24	110	100%	87.3%	−4.98
LSTM-MV vs. S-naïve (weekly)	24	110	98.2%	79.1%	−4.09
LSTM-MV vs. ARIMA	24	12	100%	100%	−16.63

**Table 6:** Feature ablation for the LSTM-MV (no interpolation), median across 110 sensors. Each row removes one feature group from the full model.

Configuration	h=8		h=24	
	RMSE	MAPE	RMSE	MAPE
Full (all features)	6.16	69.2%	4.69	80.8%
No half-day harmonic	6.25	69.2%	4.93	81.2%
No daily harmonic	6.19	69.8%	4.70	82.2%
No weekly harmonic	6.16	68.2%	4.72	80.3%
No quarterly harmonic	6.25	68.2%	4.77	80.0%
No yearly harmonic	6.17	67.7%	4.76	79.7%
No timestamp encoding	6.20	69.4%	4.80	79.8%

**Table 7:** Spearman correlations (ρ) between quality metrics and LSTM-MV RMSE, and partial correlations. A higher RMSE denotes worse performance, so a positive ρ means longer sequences are associated with larger absolute error on the higher-volume sensors they tend to occur on; the quality effect is seen most clearly in the relative (CV-RMSE) and partial analyses.

Quality Metric	h=8		h=24	
	ρ	p	ρ	p
Rank correlations				
avg_seq_len_hours	0.715	$1.8\times 10^{-18}$	0.730	$1.5\times 10^{-19}$
std_seq_len_hours	0.729	$1.8\times 10^{-19}$	0.740	$2.7\times 10^{-20}$
completeness_active	0.483	$9.2\times 10^{-8}$	0.478	$1.3\times 10^{-7}$
seq_count	−0.538	$1.3\times 10^{-9}$	−0.524	$4.4\times 10^{-9}$
Partial correlations				
avg_seq_len∣completeness	0.617	$9.0\times 10^{-13}$	0.650	$2.1\times 10^{-14}$
completeness∣avg_seq_len	−0.169	0.078	−0.213	0.026

**Table 8:** Segmented regression of CV-RMSE against average sequence length (LSTM-MV).

Parameter	h=8	h=24
Breakpoint (hours)	3.0	2.6
Breakpoint 95% CI	[1.9,3.4]	[1.9,4.9]
Slope below breakpoint	−22.3%/h	−22.0%/h
Slope above breakpoint	−0.5%/h	−0.5%/h
ΔAIC (segmented vs. linear)	+7.2	+0.4

**Table 9:** LSTM-MV median error by interpolation strategy (metrics computed on observed time steps only). Best value in each column in bold.

Interpolation	h=8 (2 h)			h=24 (6 h)		
	RMSE	MAPE	CV-RMSE	RMSE	MAPE	CV-RMSE
None (baseline)	6.16	69.2%	**56.0%**	**4.69**	80.8%	**62.1%**
Night-zero	6.08	72.6%	59.8%	5.83	76.5%	61.2%
Linear	**5.10**	65.2%	65.8%	4.76	**65.6%**	71.0%
Spline	5.31	70.8%	68.1%	4.79	72.9%	74.2%
KNN	5.66	**64.7%**	68.2%	5.90	68.2%	74.2%

**Table 10:** Random forest meta-model predicting per-sensor RMSE, and permutation importance ($\Delta R^{2}$ when a feature is permuted).

	h=8	h=24
Model fit		
OOB $R^{2}$	0.813	0.797
CV $R^{2}$ (mean ± std)	0.713±0.266	0.761±0.200
CV $R^{2}$ (5th–95th pctile)	0.077–0.924	0.435–0.924
Permutation importance		
seq_count	0.939	0.548
avg_seq_len_hours	0.107	0.241
std_seq_len_hours	0.091	0.136
completeness_active	0.029	0.047

**Table 11:** Profiles of the two sensor clusters (median values). CV-RMSE is for the LSTM-MV at h=8.

Attribute	Urban Core	Suburban
Number of sensors	28	82
Avg. sequence length (h)	5.87	2.48
Completeness (active)	0.87	0.70
Diurnal capture ratio	0.24	0.12
Mean count (ped./15 min)	55.40	11.53
Total POI count	217.7	83.2
Transit count	53.3	28.5
Distance to centre (m)	404	1894
CV-RMSE	47.7%	59.3%

## Data Availability

The data presented in this study are openly available.
